# Psychometric Properties of the Child PTSD Checklist in a Community Sample of South African Children and Adolescents

**DOI:** 10.1371/journal.pone.0046905

**Published:** 2012-10-03

**Authors:** Mark E. Boyes, Lucie D. Cluver, Frances Gardner

**Affiliations:** 1 Centre for Evidence-Based Intervention, Department of Social Policy & Intervention, University of Oxford, Oxford, United Kingdom; 2 Department of Psychiatry and Mental Health, University of Cape Town, Cape Town, South Africa; University of Stellenbosch, South Africa

## Abstract

**Objective:**

The current study assessed the basic psychometric properties of the Child PTSD Checklist and examined the structure of symptoms of posttraumatic stress disorder (PTSD) in a large sample of South African youth.

**Methodology:**

The checklist was completed by 1025 (540 male; 485 female) South African youth (aged between 10 and 19 years). The factor structure of the scale was assessed with a combination of confirmatory and exploratory techniques. Internal consistencies for the full scale and all subscales were evaluated with Cronbach’s alpha and McDonald’s omega. Validity was assessed by comparing PTSD scores obtained by children who had and had not experienced a traumatic event, and by examining associations between total PTSD scores and known correlates of PTSD.

**Results:**

Scores on the Child PTSD Checklist clearly discriminated between youth who had experienced a traumatic event and those who had not. Internal consistencies for the full scale (and all subscales) were acceptable to good and hypothesized correlations between PTSD, depression, anxiety, somatic symptoms, and age were observed. Two of the reported fit statistics for the tripartite DSM-IV-TR model of PTSD did not meet traditional criteria and further exploratory analyses revealed a four-factor structure (broadly consistent with Simms and colleagues’ Dysphoria Model of PTSD symptoms) which provided a better fit to the observed data.

**Conclusion:**

Given the continued use of the Child PTSD Checklist in South Africa, findings offer an important first step in establishing the reliability and validity of the checklist for use with South African youth. However, further evaluation of the checklist in South African samples is clearly required before conclusions regarding its use as diagnostic tool in this context can be made.

## Introduction

Posttraumatic stress disorder (PTSD) is an anxiety disorder that may occur in the aftermath of a traumatic event. According to DSM-IV-TR, PTSD symptoms can be clustered into three categories: 1) *re-experiencing* symptoms (the person may have recurrent and intrusive recollections of the trauma), 2) *avoidance* (stimuli associated with the event are persistently avoided) and *numbing* (feeling of detachment or estrangement from others) symptoms, and 3) *hyperarousal* symptoms (persistent symptoms of anxiety or increased arousal that were not present before the trauma) [1]. However, the three-factor model postulated by the DSM-IV-TR is increasingly being questioned by researchers [2] and has received relatively little empirical support [3], [4], [5].

Recent studies using confirmatory factor analysis (CFA) to examine the symptom structure of PTSD have tended to support one of two inter-correlated four-factor models [2]: a *Dysphoria* model [3] or an *Emotional Numbing* model [6]. The Dysphoria model maintains the basic DSM-IV-TR structure but the numbing symptoms and three hyperarousal symptoms (specifically trouble sleeping, difficulty concentrating, and irritability) are hypothesized to indicate a general distress factor; labelled dysphoria [3]. Thus, the four factors in the Dysphoria model are re-experiencing, avoidance, hyperarousal, and dysphoria. Support for this model has been obtained in bereaved individuals [7], survivors of sexual and physical assault [8], [9], and disaster workers [4]. In contrast, the Emotional Numbing model also retains the basic DSM-IV-TR structure but separates the avoidance and numbing symptoms into distinct clusters. The four symptom clusters in the Emotional Numbing model are thus re-experiencing, avoidance, emotional numbing, and hyperarousal. Support for this model has been obtained amongst peacekeepers [10], cancer survivors [11], military personnel [12], medical patients [5], and refugees [13]. It should be noted that differences in model fit between the Emotional Numbing and Dysphoria models are often marginal and there is little consensus as to which model provides the best description of the structure of PTSD symptoms [14], [15]. Indeed, in some studies both models are reported to fit the data well [4], [14].

PTSD symptoms are often co-morbid with anxiety, depression, and the experiencing of somatic symptoms [16], [17], [18], [19]. Additionally, there is evidence to suggest a cumulative effect of trauma exposure on PTSD symptoms, whereby multiple traumatic experiences are associated with a linear increase in PTSD symptoms [20], [21]. Relatedly, PTSD symptoms in children and adolescents tend to increase with age [22], possibly because as children get older the likelihood of experiencing a traumatic event increases [20]. Research with adults suggests that females develop PTSD twice as often as males, even if the number of lifetime stressors experienced is equivalent [23], [24], and this gender difference has also been reported in children and adolescents [25], [26], [27].

Symptoms of PTSD have been documented in child survivors of war [28], [29], [30], [31] and disasters; including floods [27], [32], earthquakes [33], [34], terrorist attacks [34], [35], tragedies [36], [37], and community violence [35], [38]. There is also evidence that PTSD symptoms in children are associated with abuse [39] and bereavement [20], [40]. South Africa has high rates of community violence and household-level abuse, and interpersonal violence is often targeted at or witnessed by South African children [41]. Additionally, an estimated 3.4 million South African children are parentally bereaved, with 65% of deaths attributed to HIV/AIDS [42]. It is perhaps unsurprising then that studies with South African youth have reported rates of trauma exposure ranging between 82% and 100%, and PTSD rates ranging between 6% and 22% [21], [43], [44].

From both a clinical and a research perspective it is important to reliably measure the distress of children who have experienced traumatic events [36]. A number of papers have established the reliability and validity of PTSD scales for use with children and adolescents in a variety of countries; including the UK, Cambodia, Croatia, Bosnia, China, and Japan [32], [34], [36], [45], [46], [47], [48], [49]. However, to date no measures have been validated for use in African contexts.

Many studies examining PTSD in South African youth have relied on the Child PTSD Checklist [50] to measure symptoms, particularly in Xhosa-speaking communities around Cape Town [20], [21], [40], [44]. The checklist is a easily administered and is explicitly child-friendly [51]. It is a 28-item self-report scale directly derived from the DSM-IV, which rates the presence (in the past month) of 17 symptoms required by DSM-IV for a diagnosis of PTSD. Prior to completing the checklist children identify the most upsetting or frightening thing that has happened to them. Items are responded to on a four-point frequency scale (*0: Not at all; 1: Some of the time; 2: Most of the time; 3: All the time*). Additionally, in South African studies the text-based checklist is accompanied by cartoons from the Levonn/Andile trauma scale, found accessible for Xhosa-speaking Cape Flats adolescents [52].

However, the psychometric properties of the Child PTSD Checklist are currently unpublished, although Amaya-Jackson and colleagues [51] report that the full scale shows excellent test-retest reliability (*r* = .91) and internal consistency (α = .82–.95) in as yet unpublished US clinical samples (children at a specialised trauma clinic or at psychiatric inpatient units). Patterns of correlations obtained between the Child PTSD Checklist, the Beck Depression Inventory (*r* = .72), and the Multidimensional Anxiety Scale for Children (*r = *.42) suggests that the Child PTSD has good convergent validity in these samples [51]. In South African studies the checklist is often administered as an outcome measure in large community samples [20], [21] and to date there is no information regarding the reliability and validity of the checklist in non-clinical samples. Psychometric properties of measures may differ between clinical and community samples and it is imperative to establish the reliability and validity of the checklist within the context where it is to be used. Moreover, the checklist was designed for use in a US context. Cultural and linguistic differences may affect the reliability of measures developed and evaluated in western samples [53], and also make it difficult to know whether measures developed in western societies truly reflect local understandings of distress and wellbeing [54].

Given the current research focus on sub-Saharan Africa and the call to scale up mental health services in low and middle income countries [55], the need for mental health measures validated for use with African samples has been emphasised by both international organisations (such as UNICEF and the World Health Organisation) as well as academics. The aim of the current study was to assess the basic psychometric properties of the Child PTSD Checklist and explore the structure of PTSD symptoms in a large South African community sample, using data collected in a previous study examining the mental health of youth living in poor urban townships of Cape Town [40]. The underlying factor structure of PTSD symptoms was assessed using a combination of confirmatory and exploratory analyses. Reliability was assessed by examining Cronbach’s alpha [56] and McDonald’s omega [57] for the full scale and all subscales. Validity was assessed by comparing PTSD scores obtained by children who had and had not experienced a traumatic event, and by examining associations between total PTSD scores and known correlates of PTSD (gender, age, symptoms of depression, anxiety, somaticism, as well as number of traumas experienced).

## Methods

### Participants

Analyses were conducted on data obtained from a sample of 1025 children and adolescents recruited in 2005 for a study exploring psychological distress amongst children in urban South Africa [40]. Participants were recruited from nine schools, 18 non-government organisations, and from door to door sampling. The study area covered deprived peri-urban settlements in Cape Town (formerly designated for black Africans under apartheid). These areas are characterised by high population density, unemployment, property crime, rape, and violent crime [58]. The sample consisted of 540 male and 485 female children, ages ranged between 10 and 19 years (*M* = 13.40, *SD* = 2.35), and the majority of participants were Xhosa-speaking (96.10%). Additional information about the sample can be found in the original Cluver et al paper [40].

### Measures

Along with the Child PTSD Checklist the following measures were administered:

#### Child exposure to community violence checklist [59]

The checklist was adapted to reflect common types of violence in South African townships, and was modified after piloting with an independent sample of local children and caregivers [40]. Community-based violence included either being a victim of, or witnessing, the Western Cape’s four most common community crimes: robbery, assault, stabbings, and shootings [58]. Household violence included witnessing domestic violence and child exposure to sexual and/or physical abuse. In the context of high levels of corporal punishment, physical abuse was defined conservatively as being hit with an object (e.g. a broomstick, switch, stick, or metal piping) likely to cause actual or potential physical harm [60]. Children could also identify other witnessed or experienced traumas. The adapted checklist provided a count of the number of community traumas, household traumas, and total number of traumas experienced by children.

#### The children’s depression inventory – short form [61]

The Children’s Depression Inventory (Short Form) consists of ten items representing a range of depressive symptoms. For each item the child is asked to choose one statement that best reflects his/her feelings. Item scores are summed to give a total depression score. In western samples the Children’s Depression Inventory (Short Form) has good reliability (α = .71–.94) [62] and is highly correlated with the full version of the inventory (*r* = .89) [61]. Internal consistency in the current sample was α = .65.

#### Children’s manifest anxiety scale – revised [63]

The Revised Children’s Manifest Anxiety Scale is a 28-item questionnaire responded to on a yes/no scale. It provides three narrow anxiety factors (physiological symptoms, worry/oversensitivity, and concentration) and a total anxiety score. The scale has been well-validated and shows good internal consistency (α = .79–.85) and test-retest reliability (*r* = .68) [63], [64]. Recently the scale has been validated for use with South African youth [65]. Internal consistency for the full scale in the current sample was α = .81.

#### Child behaviour checklist – somatic subscale [66]

The somatic subscale of the youth self-report checklist was completed by participants. This subscale contains nine statements (e.g. I feel dizzy) that are responded to on a three-point scale (*0: Not true; 1: Somewhat or sometimes true; 2: Very true, or often true*). Research using the Child Behaviour Checklist has demonstrated its sound reliability and validity [66], [67]. Internal consistency for the somatic subscale in the current sample was α = .66.

### Procedure

All measures were translated from English into Xhosa by two Masters level researchers and independently back-translated by a Xhosa-speaking research psychologist. Translated and back-translated questionnaires were cross-checked by a team of five Xhosa-speaking community health and social workers. Due to low literacy rates [68] questionnaires were administered verbally by five interviewers. Interviewers were local community health or social workers who received training in both working with children from deprived communities and the administration of standardised questionnaires. In total participation took 40–60 minutes. The design of the overall questionnaire package was assisted by a ‘Teen Advisory Group’ of 14 children. In weekend camps, children co-designed the questionnaire booklet into the style of a teen magazine, including pictures of popular music stars and cartoons.

### Ethics Statement

Ethical approval for the study was obtained from the University of Oxford, the University of Cape Town, and the Western Cape Department of Education. Informed written consent was obtained from both children and their caregivers, but other than consenting to child participation no information was collected from caregivers. Confidentiality was maintained unless children requested assistance or were at risk of significant harm and no incentives for participation were provided.

## Results

### Traumatic Experiences and PTSD Symptomatology

Administering questionnaires through interviewers resulted in minimal missing data (less than 1%). Where missing items were identified responses were imputed using the mean of responses to all other items on the scale. Total PTSD (*M = *16.12; *SD* = 14.11), depression (*M = *2.88; *SD* = 2.72), anxiety (*M* = 11.52; *SD* = 5.27), and somaticism (*M* = 5.10; *SD* = 3.58) scores were calculated by summing relevant items. Total number of traumas (*M* = 6.66; *SD* = 5.67) as well as the number of home (*M = *3.87; *SD* = 3.87; *SD = *3.65) and community traumas (*M* = 2.08; *SD* = 3.04) experienced were calculated by summing responses to the adapted Child Exposure to Community Violence Checklist. As predicted, age was significantly correlated with the total number of traumas experienced (*r* = .12) as well as the number of community traumas (*r* = .14) and home traumas (*r* = .08) experienced. Additionally, age was significantly correlated with PTSD scores (*r* = .16) and it was therefore controlled for in all gender comparisons. ANCOVAs (controlling for age) revealed no gender difference in the number of home traumas experienced [*F*(2, 1016) = .25, *p* = .621]; however, males reported experiencing significantly more community traumas (*M* = 2.38; *SD* = 3.73) than females (*M* = 1.76; *SD* = 1.84); *F*(2, 1013) = 10.04, *p* = .002, partial η^2^ = .01. The gender difference in total traumas experienced was approaching significance [*F*(2, 1007) = 2.75, *p* = .098], with males (*M* = 7.02; *SD* = 6.47) reporting more traumas than females (*M* = 6.34; *SD* = 4.63). A breakdown of the traumatic events experienced by children is provided in Table 1.

**Table 1:** Traumatic events experienced by children in the sample (as measured by the adapted Child Exposure to Community Violence Checklist).

Trauma Type	Number of Children	Proportion of the Sample
Witnessed someone being shot or stabbed	584	56%
Hit at home	500	49%
Robbed	399	39%
Domestic Conflict (adults shouting)	191	19%
Attacked	133	13%
Hit with an item likely to cause harm (e.g. broom, switch, stick, or metal piping)	112	11%
Inappropriate/uncomfortable touching	99	10%
Domestic violence	82	8%
Serious illness	67	7%
Sexual abuse	37	4%
Other (including witnessing accidents, gang fighting, communityviolence, or seeing corpses)	293	29%

Prior to responding to the Child PTSD Checklist children self-identified the most upsetting or frightening thing that had happened to them (responses were open-ended). Eight hundred and seventy-nine children (85.75%) self-identified a traumatic event. One hundred and forty-six children (14.25%) did not identify a trauma. In order to examine differences in symptomatology between children who had and had not experienced trauma, children who did not identify a trauma were asked to respond to the items with regard to their most recent disagreement with a friend. PTSD scores clearly discriminated between children who reported experiencing a traumatic event (*M* = 18.74, *SD* = 13.87) and those who had not (*M* = 2.04, *SD* = 4.98); *F*(1, 1008), 205.61, *p*<.001, partial η^2^ = .17. All further psychometric analyses were conducted on the subsample of 879 children who reported experiencing a traumatic event. An ANCOVA (controlling for age) revealed no significant differences in PTSD scores as a function of gender *F*(2, 861) = .61, *p* = .434.

### Structure of PTSD Symptoms

A confirmatory factor analysis (CFA) was conducted in order to determine whether the DSM-IV-TR model, specifying three symptom clusters (i.e. re-experiencing, avoidance/numbing, and hyperarousal symptoms) was reflected in the South African sample. All average inter-item correlations were in the.15 to.50 range recommended by Clark and Watson [69]; however item-total correlations for item 27 (“Do you wet your pants or bed by accident”) and item 28 (“Do you feel like you are tuned out or in a trance”) did not meet the.30 criteria suggested by Field [70] and these items were dropped from the analyses. Items 1, 2, 3, 4, 5, 6 7, 10, 11, and 26 were constrained to load onto a re-experiencing factor. Items 7, 8, 9, 12, 13, 22, 23, 24, and 25 were constrained to load onto an avoidance/numbing factor. Items 14, 15, 16, 17, 18, 19, 20, and 21 were constrained to load onto a hyperarousal factor. The three factors were allowed to correlate and no correlated error terms or item cross-loadings were specified.

Analyses were conducted in AMOS 16 using maximum likelihood estimation. The following fit indices were calculated: the chi square statistic (χ^2^) and χ^2^/degrees of freedom [71], the Root Mean Square Error of the Approximation (RMSEA), the Comparative Fit Index (CFI), and the Standardised Root Mean Square Residual (SRMR). The fit statistics for the DSM-IV-TR model were: χ^2^(296) = 1391 (*p*<.001), χ^2^/df = 4.70, RMSEA = .065 (90% CI = .061–.068, PCLOSE<.001), SRMR = .051, CFI = .871. The values of RMSEA and SRMR were within acceptable limits (although PCLOSE was less than.05); however, χ^2^/df was greater than the recommended criterion of three and CFI values should be at least.90 [71]. Although researchers have cautioned against rigid adherence to cut-off vales [72], given that χ^2^/df and CFI did not meet standard criteria further exploratory analyses were conducted.

Using principal components analysis (with oblique rotation, as components were hypothesised to be correlated) three factors with eigenvalues greater than one emerged. This three-factor solution accounted for 47.36% of the total variance, which did not meet the traditional 50% minimum [70]. However, an examination of the scree plot suggested a fourth factor (eigenvalue = .98) should be extracted. This four-factor solution was broadly consistent with the Dysphoria Model of Simms et al [3] (factors were labelled hyperarousal, avoidance, dysphoria, and re-experiencing) and accounted for 51.14% of the total variance (hyperarousal = 36.49%, avoidance = 6.34%, dysphoria = 4.53%, re-experiencing = 3.78%). This four-factor structure was modelled in AMOS 16 (using maximum likelihood estimation) and item loadings are summarised in Table 2. Fit statistics for this model were: χ^2^ (293) = 967 (*p*<.001), χ^2^/df = 3.30, RMSEA = .051 (90% CI = .048–.055, PCLOSE = .288), SRMR = .041, CFI = .920. Correlations between the four factors are summarised in Table 3. The χ^2^ value for the four component model was substantially reduced in comparison with the DSM-IV-TR model (424 points with three fewer degrees of freedom) and with the exception of χ^2^/df (which was just above the recommended maximum value of three) all fit indices were adequate or good. Additional analyses confirmed the unidimesionality of the four components and there were no gender differences in scores obtained on any of the four components. However, it should be noted that these fit indices are for an exploratory model extracted from the current dataset. Therefore, further confirmatory research testing this model in an *independent* sample is clearly required before conclusions regarding the generalisability of the four-factor model beyond the current sample can be made.

**Table 2:** Factor loadings obtained in the CFA of the four factor model.

Item	Hyperarousal	Avoidance	Dysphoria	Re-experiencing
1. Nightmares/bad dreams	.63**			
12. Hard to have any feelings/feel numb?	.66**			
14. Get physically upset (sweaty, shakes, heart pounding etc)	.73**			
15. Trouble falling/staying asleep	.69**			
16. Concentration problems	.66**			
17. Need to stay ‘on guard’	.64**			
18. Get jumpy/startle easily	.64**			
19. Easily annoyed or irritated	.67**			
20. Angry/upset for no reason	.64**			
21. So angry you hit/hurt someone	.55**			
7. Try not think about what happened		.69**		
8. Stay away from things that remind you about what happened		.68**		
9. Trouble remembering what happened		.67**		
13. Keep busy to avoid thinking about it		.64**		
10. Act out or repeat things			.65**	
11. Feel like it’s happening again			.65**	
22. Think you won’t grow up to be what you want			.60**	
23. Hard to have fun			.72**	
24. Hard to feel happy			.71**	
25. Feel alone			.68**	
26. Feel bad or guilty			.48**	
2. Upset when think about what happened				.73**
3. Upset when reminded of what happened				.75**
4. Go over what happened in your mind				.69**
5. See pictures of what happened in your mind				.73**
6. Worry it might happen again				.65**

** *p*<.01;

* *p*<.05.

**Table 3:** Correlations (Spearman’s rho) between PTSD, depression, anxiety, and somaticism scores, as well as total number of traumas, number of home traumas, and number of community traumas experienced.

	1	2	3	4	5	6	7	8	9	10	11	12
1) PTSD Total	–											
2) Hyperarousal	.93**	–										
3) Avoidance	.74**	.59**	–									
4) Dysphoria	.81**	.71**	.44**	–								
5) Re-experiencing	.87**	.74**	.62**	.62**	–							
6) Depression	.42**	.42**	.21**	.40**	.34**	–						
7) Anxiety	.54**	.59**	.32**	.42**	.45**	.44**	–					
8) Somaticism	.45**	.47**	.29**	.36**	.37**	.22**	.46**	–				
9) Total Traumas	.50**	.50**	.26**	.47**	.38**	.32**	.43**	.37**	–			
10) Home Traumas	.44**	.45**	.21**	.44**	.33**	.34**	.39**	.26**	.87**	–		
11) Community Traumas	.31**	.30**	.18**	.27**	.27**	.13**	.21**	.29**	.66**	.29**	–	
12) Age	.16**	.16**	.12**	.13**	.14**	.19**	.11*	.01	.12**	.08*	.14**	–

** *p*<.01;

* *p*<.05.

### Reliability and Correlates of the Child PTSD Checklist

Cronbach’s alpha (α) [56] and McDonald’s omega (ω) [57] were used to assess the reliability of the Child PTSD Checklist. Internal consistencies for the full Child PTSD Checklist, the three DSM-IV-TR symptom clusters, and the four-factor model identified through exploratory analyses were all acceptable to good, and are summarised in Table 4. Correlations between total scores on the Child PTSD Checklist and the four factors identified in the exploratory analyses, as well as information obtained from the adapted Child Exposure to Community Violence Checklist, the Children’s Depression Inventory (Short Form), Children’s Manifest Anxiety Scale (Revised), and the somatic subscale of the Child Behaviour Checklist were calculated (see Table 3). Because the distributions of depression scores, PTSD scores, somaticism scores, home traumas, community traumas, and total traumas experienced were positively skewed, Spearman’s rho is reported for all correlations. As hypothesized, total PTSD scores (as well as hyperarousal, avoidance, dysphoria, and re-experiencing scores) correlated significantly with age, total number of traumas experienced (as well as both the number of community and home traumas), total depression and anxiety scores, and somatic symptoms.

**Table 4:** Cronbach’s α and McDonald’s ω estimates for the full Child PTSD Checklist, subscales based on DSM-IV-TR, and subscales based on exploratory analyses.

	Cronbach’s α	Macdonald’s ω
Full scale	.93	.92
DSM-IV Hyperarousal	.83	.82
DSM-IV Avoidance/numbing	.79	.80
DSM-IV Re-experiencing	.84	.82
Hyperarousal (4 Factor)	.87	.83
Avoidance (4 Factor)	.71	.70
Dysphoria (4 Factor)	.81	.78
Re-experiencing (4 Factor)	.80	.77

## Discussion

Many studies with South African youth have relied on the Child PTSD Checklist to measure PTSD symptoms [20], [21], [40], [44]. The psychometric properties of the checklist are currently unpublished and this study aimed to assess the basic psychometric properties of the Child PTSD Checklist as well as examine the structure of PTSD symptoms in a large community sample of South African children and adolescents. Over 85% of children reported experiencing a traumatic event and this is consistent with previous research in South Africa [21], [43], [44].

Two of the fit indices obtained in the CFA testing the tripartite structure of PTSD proposed by DSM-IV-TR did not meet standard criteria. However, the three-factor model postulated by the DSM-IV-TR is increasingly being questioned by researchers [2] and has received relatively little empirical support [4], [5]. Further exploratory analyses revealed an interpretable four-factor solution (consisting of symptom clusters labelled: hyperarousal, avoidance, dysphoria, and re-experiencing) which showed substantially improved fit in comparison to the DSM-IV-TR model. This model was broadly consistent with the Dysphoria Model of Simms and colleagues [3] and adds further weight to criticisms of the DSM-IV-TR model of the structure of PTSD. Given that the DSM-IV-TR is currently under revision, further research clarifying the structure of PTSD symptoms is clearly needed and cross-cultural research will be invaluable in demonstrating whether models are culturally robust [2]. However, as mentioned previously, the exploratory model was extracted from the current dataset and further confirmatory research testing this model in an *independent* sample is required before conclusions regarding the generalisability of this model beyond the current sample can be made.

Internal consistencies for the full scale, the three DSM-IV-TR subscales, and the four subscales identified in the exploratory analyses were acceptable to good, and with the exception of item 27 and item 28 (which were dropped) all items met standard criteria for inter-item and item-total correlation. These findings suggest that the checklist is a reliable measure of PTSD symptoms in South African youth. Regarding the validity of the checklist, total scores clearly discriminated between children who reported experiencing a traumatic event and children who did not. Children who had experienced a traumatic event obtained significantly higher PTSD scores than children who did not and predicted correlations between total PTSD, depression, anxiety, and somatisation scores [17], [18], [19], as well as age and number of traumas experienced [20], [21], were observed. These relationships were also observed with the hyperarousal, avoidance, dysphoria, and re-experiencing subscales identified in exploratory analyses; however, unexpectedly no gender differences in PTSD symptoms were obtained (on either the full scale or the four subscales). Previous findings have reported females to be at greater risk of developing PTSD [23], [26], [27] and further research exploring this in South Africa is required. Taken together these results suggest the Child PTSD Checklist appears to be a reliable and valid measure of PTSD symptoms the South African context.

The current study did have a number of limitations. It should be noted that whilst a potential strength of the study (in terms of missing data and children’s understanding of questionnaire items), verbally administering questionnaires through interviewers is a non-standard method for administration of the Child PTSD Checklist (and the other outcome measures). Additionally, the reliabilities of the Children’s Depression Inventory (Short Form) and the somatic subscale of the Child Behaviour Checklist in the current sample were below the recommended criterion of.70. Reliably measuring internalising symptoms in children is notoriously difficult due to problems with social desirability and limitations in children’s ability to reliably report subjective states of internal distress [73] and further research examining the performance of these measures in South African children is clearly needed. Furthermore, due to constraints of the data-set test-retest reliability was not able to be assessed and future research should evaluate this. Finally, the current study was not able to evaluate the diagnostic performance of the checklist in the South African context. The current findings are limited to establishing the basic reliability, as well as discriminant and convergent validity of the checklist. Research evaluating the diagnostic performance of the Child PTSD Checklist in terms of its sensitivity and specificity is clearly required before conclusions regarding its use as a diagnostic tool in South African samples can be drawn. As noted by Amaya-Jackson and her colleagues [51], symptom thresholds may vary across populations and research identifying appropriate clinical cut-offs in South African communities should be conducted before the checklist is used for diagnostic purposes.

However, bearing these limitations in mind, given the continued use of the Child PTSD Checklist in South Africa the current findings offer an important first step in establishing the basic reliability and validity of the checklist for use with South African youth. Results reveal that the checklist shows good internal consistency and correlates predictably with depression, anxiety, age, somatic symptoms, and traumatic experiences. Additionally, our exploratory analyses revealed four clusters of PTSD symptoms that are broadly consistent with the Dysphoria Model of Simms and colleagues [3]. This four-factor model showed substantially improved fit when compared with the tripartite DSM-IV-TR model and contributes to the growing body of literature questioning the DSM-IV-TR model of the structure of PTSD symptoms. However, further confirmatory research examining this model, as well as the Emotional Numbing model of King et al [6], is clearly needed. Finally, further evaluation of the diagnostic performance of the checklist in South African samples is required before conclusions regarding its use as diagnostic tool in this context can be made.
